# Coronavirus Disease 2019 in Children

**DOI:** 10.3389/fped.2021.668484

**Published:** 2021-05-28

**Authors:** Melissa Borrelli, Adele Corcione, Fabio Castellano, Francesca Fiori Nastro, Francesca Santamaria

**Affiliations:** Section of Pediatrics, Pediatric Pulmonology Unit, Department of Translational Medical Sciences, Università di Napoli Federico II, Naples, Italy

**Keywords:** COVID-19, novel coronavirus, SARS-CoV-2, children, adolescents, chest imaging, drugs, therapy

## Abstract

Since its appearance in Wuhan in mid-December 2019, acute respiratory syndrome coronavirus 2 (SARS-CoV-2) related 19 coronavirus disease (COVID-19) has spread dramatically worldwide. It soon became apparent that the incidence of pediatric COVID-19 was much lower than the adult form. Morbidity in children is characterized by a variable clinical presentation and course. Symptoms are similar to those of other acute respiratory viral infections, the upper airways being more affected than the lower airways. Thus far, over 90% of children who tested positive for the virus presented mild or moderate symptoms and signs. Most children were asymptomatic, and only a few cases were severe, unlike in the adult population. Deaths have been rare and occurred mainly in children with underlying morbidity. Factors as reduced angiotensin-converting enzyme receptor expression, increased activation of the interferon-related innate immune response, and trained immunity have been implicated in the relative resistance to COVID-19 in children, however the underlying pathogenesis and mechanism of action remain to be established. While at the pandemic outbreak, mild respiratory manifestations were the most frequently described symptoms in children, subsequent reports suggested that the clinical course of COVID-19 is more complex than initially thought. Thanks to the experience acquired in adults, the diagnosis of pediatric SARS-CoV-2 infection has improved with time. Data on the treatment of children are sparse, however, several antiviral trials are ongoing. The purpose of this narrative review is to summarize current understanding of pediatric SARS-CoV-2 infection and provide more accurate information for healthcare workers and improve the care of patients.

## Introduction

Acute respiratory syndrome coronavirus 2 (SARS-CoV-2) is the first global pandemic of the twenty-first century. It soon became surprisingly clear that children were infected far less frequently than adults and had less severe symptoms (1–3). However, it was later found that also children and adolescents may experience severe clinical symptoms and even an unfavorable outcome (1). Indeed, in the USA in which the infection has not been contained, large proportions of children have required acute or critical hospital care (4). In this narrative review, we explore the epidemiology, clinical presentation, outcomes, and laboratory and chest imaging features of pediatric 19 coronavirus disease (COVID-19). We also explore why the pediatric population is generally far less at risk of developing the disease than the adult population. Lastly, we review the rapidly growing data on the treatment of children with COVID-19.

## Epidemiology

According to WHO data available at time of writing, the cumulative number of reported cases of COVID-19 is just under 100 million, with over 2 million deaths globally since the start of the pandemic (5). The prevalence of the disease remains very high in some areas of the world including the Americas, Europe, and South-East Asia, and fairly low in Africa, the Western Pacific, Australia, and New Zealand (5).

**Figure 1:**
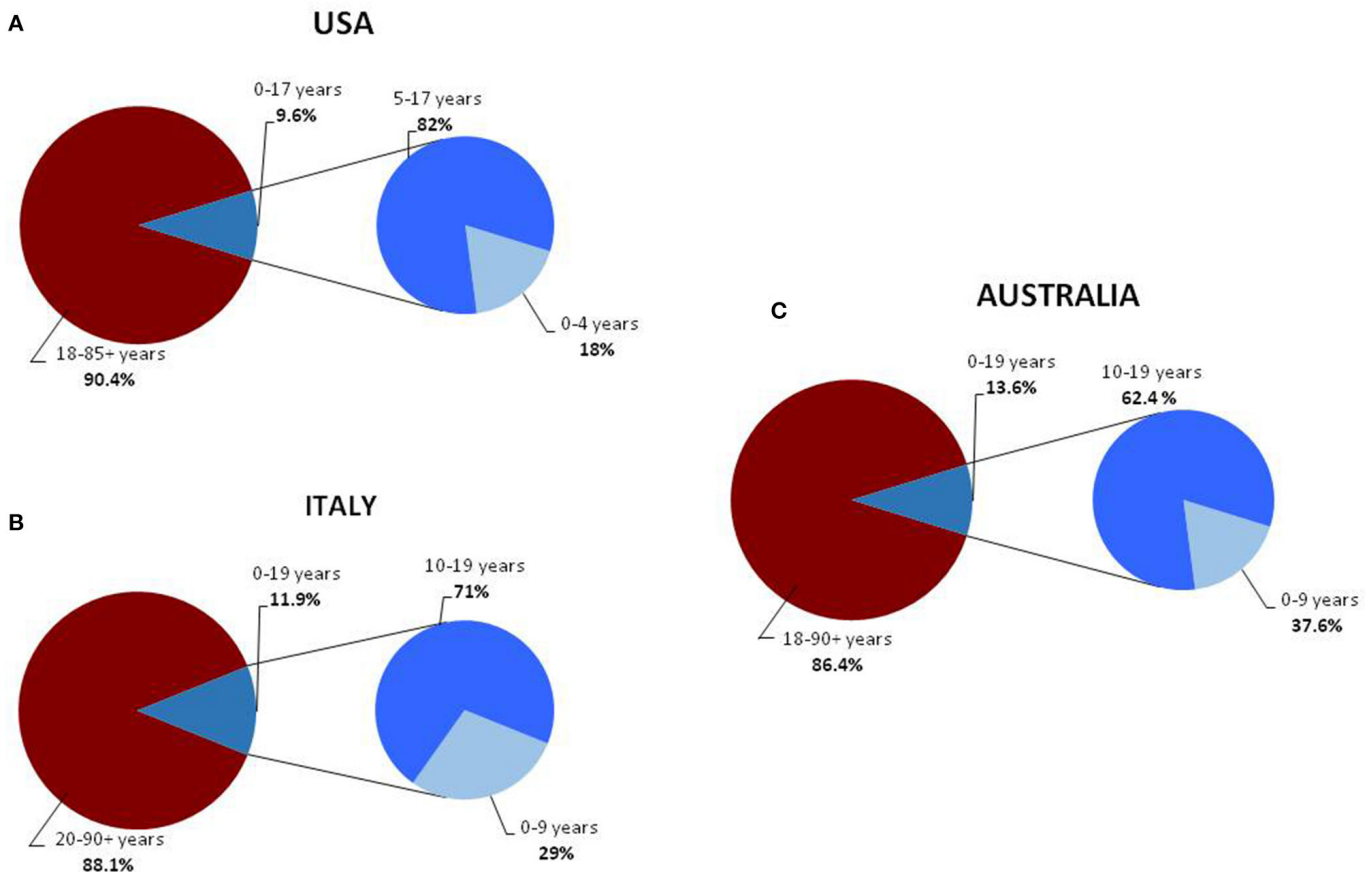
Distribution of COVID-19 cases, in the general and pediatric populations of the USA **(A)** (14), Italy **(B)** (16), and Australia **(C)** divided into age groups (17).

Childhood COVID-19 cases occurred early in the outbreak. The first pediatric case, which was reported on January 20, 2020, was a 10-year-old boy from Shenzhen, China, whose family had visited Wuhan City (6). Soon after, a retrospective study summarized the main clinical manifestations of the SARS-CoV-2 infection in children hospitalized because of respiratory symptoms occurring between January 7 and 15, 2020 (7). Between January and November, 2020, new pediatric cases were recorded in various parts of the world (8–17) (Table 1). Initially, the percentages of infections were lower in children than in adults. Sustained social interventions such as quarantines and infection control can be effective in interrupting transmission chains (18, 19). In fact, strict mandatory quarantines and infection control measures halted the increase in pediatric COVID-19 cases in March 2020 (1). Notably, countries where restriction measures have not been implemented or have been adopted for short periods of time, the spread of infection has not been contained. In the USA and in Europe, the case notification rate increased rapidly in September and October, 2020, and the number of pediatric cases was higher than at the start of the pandemic (20, 21) (Table 1). The higher percentage of pediatric cases is probably due to an initial underestimation of the prevalence of COVID-19 in children. In fact, in the early phases of the outbreak, children were rarely tested for the virus as they had no or only mild symptoms that did not require hospital admission. In Australia, which has high rates of testing, the percentage of pediatric cases on a low total number of confirmed cases are similar to rates in the USA and Europe (20). Despite the substantial increase in cases among children and adolescents in the USA, Italy, and Australia, the percentages of pediatric cases remain far below those of adults (14, 20, 21) (Figure 1). Data from various areas of the world indicate that the median age of COVID-19 pediatric cases ranges from 5 to 7 years, with the exception of the USA where the median age of infection is 11 years, with more cases in the 10–19 year age group than in the 0–9 age group and a mild preponderance of males (1, 2, 21).

**Table 1:** Prevalence rate of pediatric COVID-19 from January to November, 2020 in the world.

	**January**	**February**	**March**	**April**	**November**
China	First pediatric case reported (6)		–	–	–
	0.6% (8)	2.4% (9)−3.4% (10)			
Europe	–	Only 3 pediatric cases reported (11)	–	3.6% (12) In Italy 1.9% (15)	– In Italy 11.7% (16)
United States of America	–	–	First pediatric case reported (2)	1.7% (2)	9.6% (14)
Australia	–	–	–	4% (13)	13.6% (17)

The childhood form is quite mild, and the case fatality rate is much low. A large study published online on March 16, 2020 that enrolled 2,143 pediatric patients from the Hubei Province and border areas of China, reported only one death (1). Two months later, three deaths were reported in 2,572 USA pediatric cases, which equals a mortality rate of 0.12% (2). On April 8, 2020, the European Center for Disease Prevention and Control reported 3 pediatric deaths (12). On May 8, 2020, the Italian case-based surveillance system of confirmed SARS-CoV-2 infections reported four deaths on a total of 3,836 pediatric patients (mortality rate 0.1%) (22). Although, the number of infected children has increased, the mortality rate is lower now than at the beginning of the pandemic (20, 23, 24). In November 2020, there were 141 deaths in the USA pediatric population (50 in children aged 0–4 years, and 91 in children aged 5–17 years), with a total mortality rate of 0.016% (141 on 881,250 infected children) (23). In the same month, the Italian National Institute of Health recorded 9 deaths of children under the age of 9 years and 3 deaths in the 10–19 year age group (12 on 95,477 infected children, mortality rate 0.012%) (24). No deaths in were reported in the Australian pediatric population (20).

The most common source of infection of children and adolescents is a parent or sibling, followed by contact with a person outside the family or an unknown subject (25). In a study of 40 children with COVID-19, symptoms started after, or were concurrent with the presence of infected adult household contacts, which suggests that children were probably not the source of infection (26). Moreover, there is growing evidence that children, particularly school-age children, are far less susceptible to infection (27) and less frequently drivers of SARS-CoV-2 transmission than adults (28). SARS-CoV-2 can be vertically transmitted from an infected mother to her infant, albeit rarely (29). Only three of 91 (about 3%) neonates born to mothers infected with SARS-CoV-2 showed elevated levels of IgM against the virus in the serum at birth (29).

It can be transmitted through the placenta and can thus be detected by quantitative, real-time, reverse-transcription polymerase chain reaction (RT-PCR) in amniotic fluid, breast milk, cord blood, and newborn throat swab (30, 31). Although neonates may test positive for SARS-CoV-2 shortly after birth (32), their nasopharyngeal swabs become negative within a few days (33). Therefore, it is conceivable that the isolation of viral RNA from the upper airways might be less reliable in identifying cases of vertical transmission than in identifying cases of direct respiratory tract infection. Serial testing of stool specimens in the first day of infection might increase the overall diagnostic yield. Therefore, pregnant women should be screened for COVID-19 and undertake strict infection control measures. Infected mothers should be quarantined, and neonates at risk of COVID should be closely monitored (34).

## Pathogenesis and Possible Protective Mechanisms Against SARS-CoV-2 Infection in Children

Historically, SARS-CoV and the Middle East Respiratory Syndrome (MERS) viruses caused the outbreaks of 2003 and 2012, respectively (35). Both SARS-CoV and SARS-CoV-2 bind to angiotensin-converting enzyme 2 (ACE2) that is part of the renin angiotensin system (RAS) (36). Activation of RAS starts with the production of renin by the renal juxtaglomerular apparatus. Renin converts angiotensinogen into angiotensin 1, which is then transformed into its active form angiotensin II by the angiotensin converting enzyme (ACE) (37, 38).

ACE2, which promotes the release of several vasoactive anti-inflammatory peptides (39), is the functional receptor used by SARS-CoV and SARS-CoV-2 to enter the heart, intestine, kidney, and the endothelial and type II alveolar host cells through the viral spike (S) protein (40). The S protein requires cleavage by proteases to bind to the cell membrane and enter the host cell. Several host proteases can cleave the S protein of SARS-CoV-2, including transmembrane protease serine protease-2 (TMPRSS2) and cathepsin L protease. TMPRSS2 is essential for viral entry and spread in the host infected by SARS-CoV-2 (41). The subsequent endocytosis of the SARS-CoV-2 virus linked to the ACE2 receptor causes a reduction of the ACE2 molecules expressed on the cell surface (39). Therefore, SARS-CoV-2 limits the activity of the ACE2-mediated metabolic pathway and thus promotes the development of inflammation in the lungs and myocardium (42, 43). An animal model of the acute respiratory distress syndrome (ARDS) provoked by SARS-CoV and SARS-CoV-2 infection showed that down-regulation of ACE2 causes an imbalance of ACE/ACE2 activity and results in accumulation of angiotensin II and in an increase of the pro-inflammatory response (44).

The burden of COVID-19 morbidity, and certainly mortality, lies chiefly in the adult population. Children are less severely affected than adults, but why this is so remains to be established. A reasonable theory argues that because ACE2 reduces inflammation, a decrease in pulmonary ACE2 activity might lead to lung inflammation (42), and because the lung expression of ACE2 and TMPRSS2 progressively increases with age (45), it might be more difficult for SARS-CoV-2 to enter the airway cells of children and replicate. Notably, the 2003 SARS epidemic resulted in a high fatality rate of infected adults, but not of children below the age of 12 years, and the most plausible explanation of this is that children and adolescents have a relative resistance to SARS-CoV (46). Moreover, the fact that SARS-CoV and SARS-CoV-2 probably enter host cells *via* the same mechanism may explain why pediatric COVID-19 is less severe than the adult form. It has been demonstrated that the MERS virus enters host cells *via* a way other than by SARS-CoV and SARS-CoV-2, yet most children with MERS were asymptomatic or had only cold-like symptoms (47, 48). However, in contrast to animal models, no significant differences were found in ACE and ACE2 expression in the BAL of newborns, children or adults with ARDS, which indicates that age is not associated with changes in the human pulmonary RAS (49). Finally, it was recently ruled out that the expression levels of viral-entry associated genes (including ACE2 and TMPRSS2) might contribute to the milder symptoms in the pediatric population (50). These findings call into question the concept that ACE and TMPRSS2 expression play a role in pediatric COVID-19 (51).

The milder symptoms in children may also reflect differences between the adult and pediatric immune systems. In fact, children develop a very effective protective mechanism against infection designated “trained immunity.” Trained immunity is a long-term epigenetic reprogramming of innate immune cells resulting from the administration of mandatory vaccinations and Bacillus Calmette-Guérin (BCG) vaccination or from frequent viral respiratory tract infections that might help to protect children against SARS-CoV-2 (52). However, it is unclear whether trained immunity provides a permanent immune memory, even though it protects against the most common pathogens (53). It is unlikely that trained immunity from BCG at birth could explain why children have a less severe COVID-19 course than adults as many countries do not include this vaccine in national schedules, and COVID-19 in newborns is mild worldwide.

Early immune responses to viral infections in children are characterized by a higher activation rate of the interferon (IFN)-related innate immunity response and by a higher level of Th1 and natural killer cells vs. adults (54). Indeed, SARS-CoV-2 antagonizes type I IFN signaling, which is already impaired in the elderly, so that a rapid replication of the virus develops in the early stages of viral infection in adults (55). During SARS-CoV-2 infection, the down-regulation of type I IFN signaling could result in a decreased Th1 response, as well as in increased Th2 and adaptive immunity responses, all of which play a central role in the development of late complications of adult COVID-19 (56). Adults with severe COVID-19 have increased levels of the inflammatory interleukins (IL)-2, IL-6, IL-7, IL-10, and tumor necrosis factor-α that constitute the “cytokine storm” (55). In fact, liver transplant children who receive immunosuppressive treatment have a low production of inflammatory cytokines and reduced activation of adaptive immunity, and despite being infected by SARS-CoV-2, are not at increased risk of severe lung disease (57). Also Th2 eosinophilia and the associated hypersecretion of IL-13, IL-5, and IL-4 protect against SARS-CoV-2 infection. A study of the genetic and biological regulatory mechanisms governing ACE2 and TMPRSS2 expression showed that, *via* IL-3, Th2 inflammation upregulates a network involving TMPRSS2 and reduces ACE2 expression in the airways, while the type 1 IFN response to respiratory viruses increases ACE2 expression (58). Having eosinophilia, a condition very common in children and adolescents with atopic asthma, or simply with atopy, does not seem to increase the risk of a poor outcome of SARS-CoV-2. However, current data are limited and the question remains open.

It may also be speculated that mandatory vaccinations in children provide cross-protection by eliciting an enhanced immune response against other respiratory pathogens including SARS-CoV-2. Indeed, a 2007 study of mice immunized with various vaccines showed no cross-reactivity against SARS-CoV-2 (59), and the same could apply to SARS-CoV-2, but this remains to be established. Most children with human coronavirus infection develop a protective immune response against other respiratory viruses that are equally common in early life (60). It has been speculated that common coronaviruses and SARS-CoV-2 share structural similarities, for instance, they share the viral S proteins, and consequently, the adaptive immune response against coronaviruses could provide protection against SARS-CoV-2 (61). Hence, a high recurrence of airway infections in children, combined with non-specific effects of mandatory vaccinations, may protect also against SARS-CoV-2 (62). In this context, a recent study suggested that pre-existing T cell-mediated cross-reactivity with SARS-CoV-2 cannot solely be explained by prior exposure to human coronaviruses, thus questioning the concept that cross-reactivity helps to reduce susceptibility to infection and thereby result in a less severe infection in children than in adults (63). Other possible protective mechanisms are the differences in endothelial damage due to age-related changes in the protein concentration of the coagulation system. In addition, quantitative and almost certainly qualitative differences occur in the hemostatic system with age, and these differences probably have a substantial impact on the epidemiology of thromboembolic diseases in children (64). These differences might help to decrease the risk of thromboembolic and/or hemorrhagic events in neonates and children with SARS-CoV-2. Lastly, children, especially toddlers, are considered to have healthier airways than older people because they spend less time outside, usually undertake fewer international journeys, have not been exposed as much as adults to cigarette smoke and pollution, and have fewer underlying conditions, all of which may help to reduce the risk of severe COVID-19 (65).

**Figure 2:**
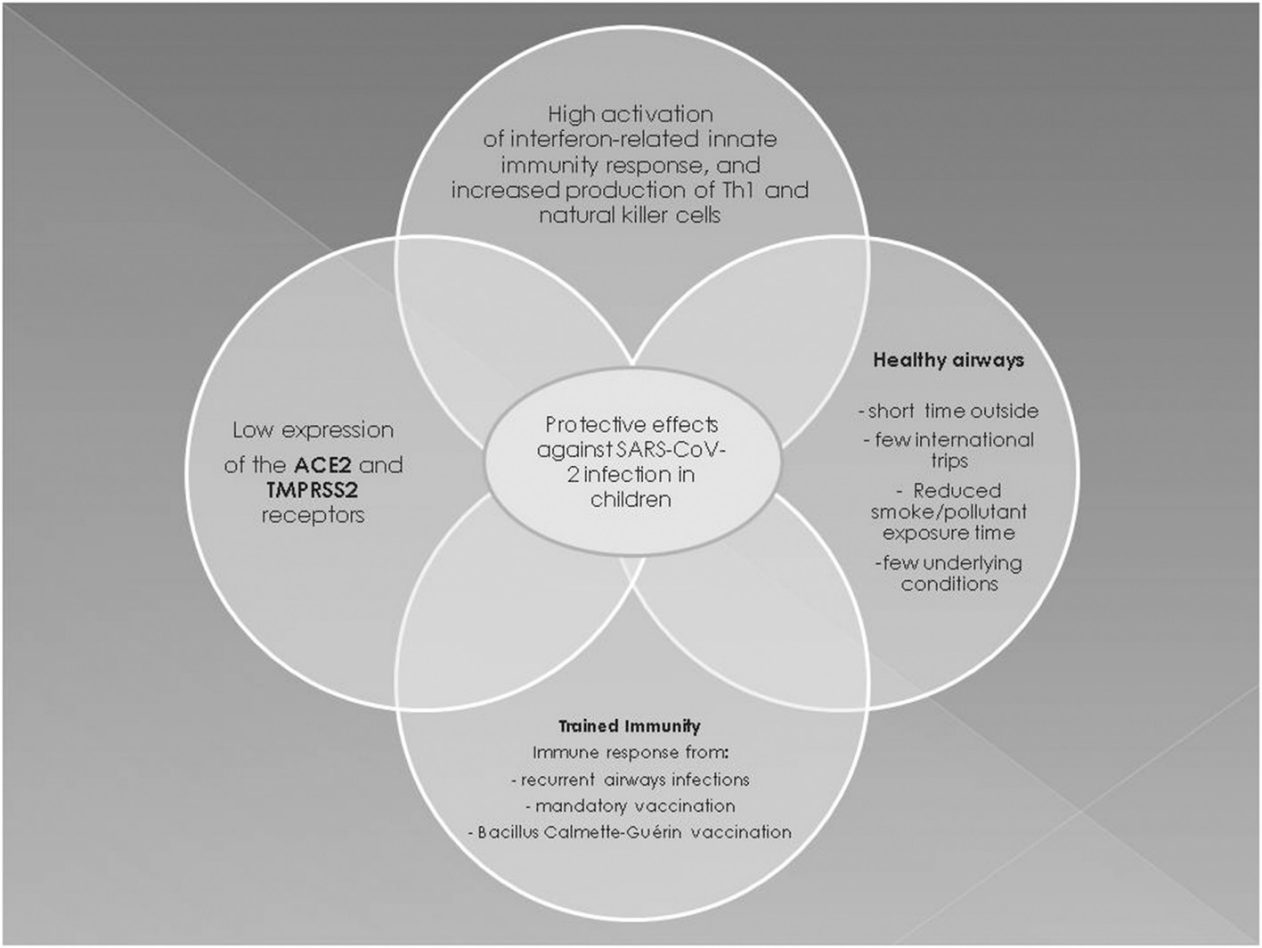
Possible protective mechanisms in the pediatric population against SARS-CoV-2 infection.

In conclusion, multiple factors are involved in the pathogenesis of less severe COVID-19 in children (Figure 2). The prevailing hypotheses may be difficult to confirm and only postmortem histopathological changes might provide insights into the pathogenesis of pediatric COVID-19 (56).

## Clinical Features of Pediatric COVID-19

**Figure 3:**
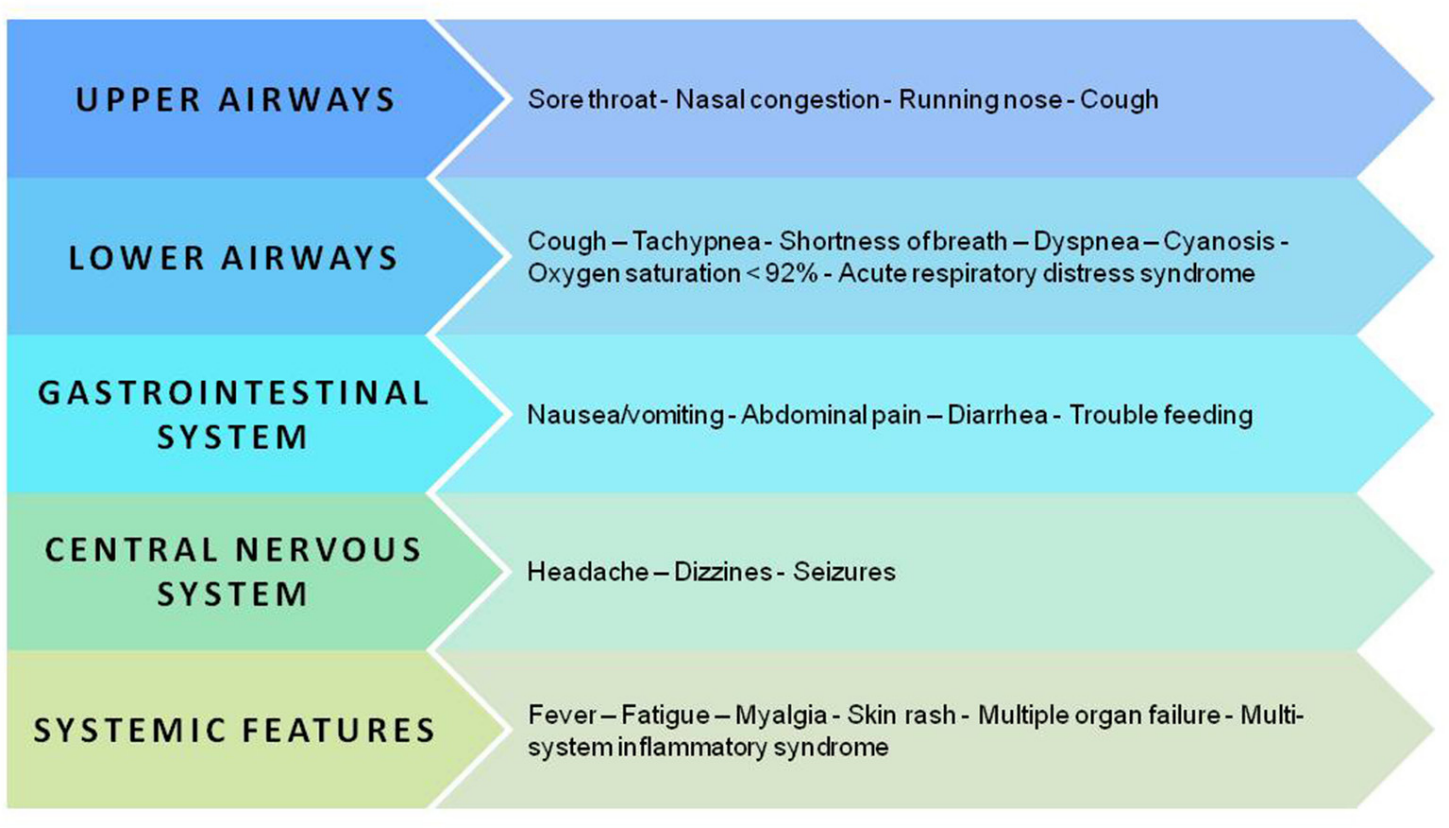
Key manifestations and organ and apparatus involvement in the pediatric population with SARS-CoV-2 infection.

Initially, the data about pediatric COVID-19 were reassuring as most children had mild disease or were asymptomatic, and there were fewer critical cases and fewer deaths than among adults (1, 66, 67). More recent data, collected from a very large sample, confirmed that children diagnosed with COVID-19 have an excellent prognosis even though longitudinal studies are required to verify this finding and to eventually explain why some patients develop severe inflammation and even multiorgan failure (68–70). Figure 3 summarizes the clinical manifestations of COVID-19 in children.

After an average incubation period of between 3 and 7 days (range, 1–14 days), children may or may not develop symptoms (21). The absence of clinical symptoms in children has been reported in variable proportions ranging from 4.4% (1) to 14–19.3% (69, 70), and even 39% (22). These differences might reflect the different enrollment criteria of study populations. The first reports from China mainly concerned children with a diagnosis of COVID-19 based only on the presence of clinical manifestations (1). More recent data from Italy refer to a higher percentage (~75% in the 2–6 years age group) of asymptomatic cases among children and adolescents (15). This percentage might be explained by the large number of subjects who were tested for screening purposes or contact tracing in September, October, and November, 2020, in Italy.

When symptoms develop, fever, usually below 39°C (53–59%), and cough (48–56%) dominate the clinical scenario (69–71). Other less frequent clinical features are upper respiratory symptoms and/or lower respiratory airway symptoms (69–74) (Table 2). Dyspnea is closely associated with the development of the most severe or critical manifestations of the disease, in particular acute respiratory distress syndrome (ARDS) (72). Up to 25% of cases may develop gastrointestinal symptoms, namely nausea, vomiting, and diarrhea, which may result in decreased intake of fluids and solids (69, 70). Additional symptoms are fatigue and myalgia, headache, and dizziness, seizures in the absence of any underlying seizure disorder and skin rash (69, 70, 73, 74). Symptoms and signs are rarely isolated in COVID-19 children (21, 75, 76). In fact, fever is frequently associated with cough or diarrhea or upper respiratory tract infections (URTI) (73) (Table 2).

**Table 2:** Clinical features of SARS-CoV-2-infected children.

**Signs and symptoms**	**Percentages (%) of cases**
Fever	53–59% (69, 70)
Cough	48–56% (69, 70)
Fatigue, myalgia	7.6% (71)−18.7% (70)
Chest pain	2.4% (74)
**Upper respiratory tract**	
Sore throat	18.2% (70)
Nasal congestion and running nose	10–20% (70)
Pharyngeal erythema	3.3% (70)
**Lower respiratory tract**	
Shortness of breath; dyspnea	9.5% (74)−11.7% (70)
**Gastrointestinal system**	
Nausea	5.4% (70)
Vomiting	5.4% (70, 74)−6.4% (71)
Diarrhea	6.5% (70)−13.1% (74)
Abdominal pain	6.5% (70)
Decreased oral intake	1.7% (70)
**Central nervous system**	
Headache; dizziness	4.3% (70)
Seizures	
Non-febrile	1.8% (74)
Febrile	1.2% (74)
**Skin**	
Rash	0.25% (70)
**Concurrent symptoms/signs at presentation**	
Fever and cough	8% (73)
Fever and diarrhea	6.4% (73)
Fever, cough, and vomiting	2.4% (73)
Fever and nasal symptoms	2.4% (73)
Fever, nasal symptoms, and cough	2.4% (73)
Fever, pharyngitis, and cough	1.6% (73)
Fever, pharyngitis, and diarrhea	1.6% (73)

Pneumonia has not been reported in children with a mild SARS-CoV-2 infection who usually present with fever, cough and/or fatigue due to URTI (22, 77). In moderate and severe cases of COVID-19, pneumonia can present with multiple associated symptoms, i.e., fever, cough, myalgia, signs of respiratory distress, cyanosis, neurological signs and symptoms, trouble feeding and signs of dehydration and hypoxemia (77). The presence of lower respiratory tract infection signs or symptoms at presentation was observed in the 25% of children (21). The assessment of imaging features combined with clinical and laboratory findings facilitates the diagnosis of COVID-19 pneumonia. Intensive care should be considered for children with severe, unremitting symptoms and signs that fulfill the criteria of critical illness with respiratory failure (78). ARDS rarely occurs (5–8%) in children affected by COVID-19 (21, 74). Pediatric ARDS is acute respiratory failure characterized by symptoms of severe hypoxemia and radiographic changes that occur within few days of a known clinical insult (79). Other acute severe and critical disease pictures include shock, encephalopathy, myocardial injury or heart failure, in addition to coagulation dysfunction and acute kidney injury (22, 72). Organ dysfunction can be seriously life threatening (1).

Most children with COVID-19 have an uneventful course. However, an inflammatory scenario similar to Kawasaki disease (KD) or toxic shock syndrome, now qualified as the COVID-19-associated multi-system inflammatory syndrome in children (MIS-C), has been identified within 2–6 weeks after acute SARS-CoV-2 infection (80). MIS-C is a rare post-COVID-19 complication (incidence 0.14%) (70). Symptoms and signs include unremitting high fever, abdominal pain, vomiting, diarrhea, rash, conjunctivitis, mucocutaneous disease and hypotension, and only few cases require admission to an intensive care unit (ICU). Initially considered an atypical form of KD (81–83), it has become later evident that shock, gastrointestinal symptoms, and coagulopathy, which are rarely seen in classic KD, are prominent features of this unique syndrome and are frequently associated with elevated levels of ferritin, D-dimers, troponin, procalcitonin (PCT), and C-reactive protein (CRP) (84, 85). In addition, coronary artery abnormality and ventricular dysfunction were evident at cardiac imaging (86). Future investigations on MIS-C should aim to correlate primary COVID-19 signs and symptoms with MIS-C incidence and presentation to identify useful predictive factors for developing this potentially life-threatening condition.

Severe and critically ill children requiring hospital admission or intensive care are rarely seen (1, 55, 67). Indeed, most pediatric studies report a low incidence of hospitalization and observation or treatment in ICUs (70, 73). In Italy, the number of hospitalized children with COVID-19 was 511 on a total of 3,836 infected pediatric patients (hospitalization rate 13%), of whom 18 (3.5%) were admitted in ICU (22). Other studies reported higher hospitalization (up to 68%) and ICU admission (between 8 and 19.5%) rates (21, 69, 70, 76, 87). We speculate that this may reflect differences in Coronavirus testing eligibility. In fact, in many countries, priority testing is given to hospitalized children, children with symptoms and signs of COVID-19, and to children considered severely ill or with comorbidities and a high risk of complications. This approach might result in an overestimate of the percentage of children who require hospitalization and/or treatment in an ICU. Other reason includes an increased awareness of possible severe COVID-19 children among physicians, with the use of hospitalization as an observational-preventive means. Importantly, a higher risk of disease severity has been reported in adolescents and in neonates, infants and pre-school children. This suggests an age distribution of COVID-19 severity that is difficult to explain (1, 21, 22, 72, 74).

In the pediatric population, the factors associated with ICU admission, which indicates more severe or critical conditions, are male gender (about 60%), pre-existing medical conditions (36–50%), of whom immunosuppression, respiratory, cardiovascular, and oncologic disorders accounted for the majority (65%), signs or symptoms of lower respiratory tract infection yet at presentation (73%), viral co-infection (15%), and radiological changes suggestive of pneumonia or ARDS (24–30%) (21, 22, 70, 72–74, 88). In a cohort of adolescents in New York, ARDS occurred in 30% of patients admitted to an ICU, a high proportion (85.6%) of whom required invasive mechanical ventilation (72). Indeed, even in younger children, invasive mechanical ventilation is mandatory in case of ARDS (89). Fortunately, ARDS is associated with a very low mortality rate (0.09%) (70).

Very few cases of neonatal COVID-19 have been described to date, and most infected newborns had mild or no symptoms (90). Cases of severe complications, including pneumonia, were sporadic (3/33 newborns with mothers with COVID-19) and may have been the consequence of prematurity or asphyxia or sepsis rather than of SARS-CoV-2 infection (33). Currently, the WHO recommends that mothers with suspected or confirmed COVID-19 be encouraged to initiate or continue breastfeeding (91). In fact, no case of COVID-19 transmission through breastfeeding has been reported. Moreover, the risk of COVID-19 infection is low in infants, and the infection is typically mild or asymptomatic, while the consequences of not breastfeeding and separating mother from child can be significant. Strict adherence to infection prevention and control measures is essential to prevent contact transmission between suspected or confirmed COVID-19 mothers and their infants and young children (92). In general, all individuals who are in close contact with newborns must take precautions including hand hygiene.

## Laboratory and Imaging Features of Pediatric COVID-19

To-date, only a few small sized observational studies have evaluated laboratory findings in children with COVID-19, and most data derive from case reports or case series. The most frequently reported laboratory findings in children with COVID-19 are white blood cell and platelet count, erythrocyte sedimentation rate (ERS), C-reactive protein (CRP), urea, creatinine, alanine aminotransferase, and aspartate aminotransferase (73). The white blood cell count was found to be within normal values (mean 7.1 × 10^3^/μl; normal range 4–12) (70), and an elevated white blood cell count was seen in only 10.4% of COVID-19 children (73). Neutrophils were mildly decreased (44%; normal range, 54–62%), while lymphocytes were marginally elevated (40%; normal range, 25–33%) (70). The platelet count was normal in most studies (mean 272.7 × 10^3^/μl; normal range 150–450) (70). Thrombocytosis and thrombocytopenia have also been reported, albeit rarely (73). Liver and renal function tests were normal in most reports, while serum levels of inflammatory markers (CRP, D-dimer, PCT, ERS, creatine kinase, lactate dehydrogenase, and IL-6) exceeded the mean (70, 73).

Yoon and colleagues conducted a systematic review of the laboratory characteristics in symptomatic and asymptomatic children with COVID-19 (93). CRP levels were high approximately in 23% of symptomatic and in only 5.7% of asymptomatic cases, respectively. Symptomatic children, especially those under the age of 10 years, were more likely to have a significantly higher lymphocyte count than asymptomatic children (14% of symptomatic patients vs. 0% of asymptomatic patients) and high PCT levels (54.5% symptomatic vs. 12.5% of asymptomatic patients), although the latter was not significantly different (93).

Laboratory findings differed between patients with moderate symptoms and/or signs and those with severe symptoms. Moreover, the findings in children with severe COVID-19 often overlapped those reported in adult patients (94). Children with mild COVID-19 had relatively few laboratory abnormalities, the most frequent being a decreased neutrophil count. Notably, CRP, PCT, and LDH levels were not as frequently elevated in mild COVID-19 cases as it would be expected (94). Children aged <1 year with mild COVID-19 showed an increased leukocyte counts, and LDH and liver enzyme levels (94). The latter finding may reflect a higher viral burden in this age group, given that infants have been reported to have a higher risk of developing severe disease than older children (94). However, no significant differences were observed between healthy controls and children affected by COVID-19 in terms of serum inflammatory markers.

Elevated leukocyte levels were less frequent than expected in children with severe COVID-19. Indeed, only a few children (25%) had elevated counts (95). Increased and decreased lymphocyte levels were observed at an equal frequency (18.7%), and acute-phase reactants (CRP, PCT, and LDH) were commonly elevated. Two studies reported high levels of IL-10, IL-6, and interferon-gamma (IFN-gamma) in nine children, while other cytokines were normal (95). A trend toward elevated CK-MB, D-dimer, and prothrombin time was also observed (94). Platelet counts were significantly lower in children with COVID-19-related ARDS than in children without the ARDS-related form of the disease (169 × 10^3^/mL vs. 216 × 10^3^/mL). Serum levels of CRP, PCT, LDH, pro-B type natriuretic peptide, and IL-6 were frankly elevated in patients with ARDS, but only IL-6 levels differed significantly from levels in children without ARDS (78.7 vs. 16.4 pg/mL) (72).

Lymphopenia was more severe in children with COVID-19-associated MIS-C than in children without MIS-C (11 vs. 42%) (70). Serum LDH and D-dimer levels were elevated in MIS-C patients, but no difference was found in platelet count or in liver function markers. Children with MIS-C had low levels of circulating CD16^+^ CD56^+^ natural killer cells (70). The latter patients also had significant cardiac dysfunction and high levels of troponins. Therefore, regular cardiac troponin monitoring in hospitalized children with COVID-19 may help to identify ongoing or imminent cardiac injury.

Thoracic imaging with chest X-ray (CXR), lung ultrasound (LUS), and computed tomography (CT), generally and high resolution CT are key tools for pulmonary disease diagnosis and for the management of COVID-19 in children and adolescents. Although CXR is mostly preferred for the diagnosis of pneumonia in children, few studies on its use in children with COVID-19 have been published, and, moreover, findings are often reported using non-standard terminology (96, 97). Typical CXR features include increased central peri-bronchovascular markings (also known as perihilar peribronchial wall thickening) and airspace consolidations (96). A unilateral increase in density is commonly detected at CXR, the lower zones being predominantly affected without any significant changes between the right and left lung (97). An interstitial pattern is less frequently found, and pleural effusion, pneumothorax and atelectasis are uncommon (96, 97).

**Figure 4:**
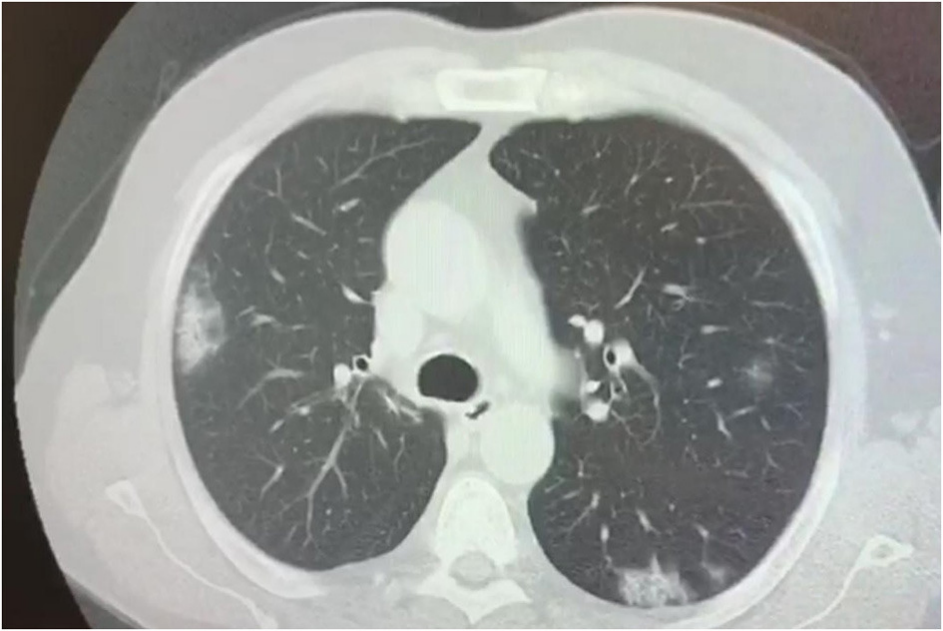
Chest CT scan showing bilateral consolidations in a 17 year old boy affected by SARS-CoV-2.

Several studies have described the chest CT findings of pediatric COVID-19 patients, however, most of the abnormalities are non-specific (98–100). Rates and numbers of children with COVID 19 who underwent chest CT scan was extraordinarily high at least in several initial reports from China, as almost all patients, also those with early disease, were ordered CT to rule out chest involvement (101). Children showed similar, but less severe CT changes than adults, which probably indicates a milder inflammatory response induced by SARS-CoV-2 (Figure 4). Furthermore, normal CT scans have been reported in a high number of children (up to 77%) (100). Ground-glass opacities (GGO) and consolidations are the most frequently observed CT lung changes in pediatric COVID-19. They can be isolated or combined, and they occur mainly in the peripheral or posterior and sub-pleural areas (102). In children with COVID-19, GGO appear to be more often unilaterally distributed, be more localized in extent, have a lower accentuation, and less lobular involvement (103, 104) than in adult patients. Usually, CT findings correlate with disease activity and severity in children with COVID-19 pneumonia, although the association between imaging severity and clinical symptoms has not been definitively established (100). More diffuse CT changes, including bilateral consolidations have been observed in children with co-infections (105), in those below the age of 3 years (106), and in those requiring intensive care (66). Follow-up CT has also been used to assess recovery from lung abnormalities, the progression of consolidations into GGO, and the presence of residual lung fibrosis (101). In conclusion, the CT findings in children with Covid-19 are usually mild and non-specific, and, at follow-up, most children have improved or normal imaging, which shows that chest CT imaging adds little to the further management of pediatric patients and therefore its usefulness is in question (107, 108).

During the last decade, LUS has been increasingly used in all age groups to diagnose several lung disorders, mostly pneumonia, in both adults and children (109, 110). It is a promising non-invasive, bedside technique, and can be recommended in the follow-up of Covid-19 infection. Very few clinical studies have been conducted using LUS in pediatric patients with COVID-19 (111, 112). This procedure reveals the typical bilateral pattern of diffuse interstitial lung involvement, subpleural consolidations and, less frequently, pleural effusion (109, 113, 114). Interestingly, LUS has been used to support the clinical suspicion of SARS-CoV-2-related pneumonia and in the longitudinal follow-up of infected children (111).

An important and widely debated issue is when and which chest imaging tool should be used in the management of pediatric COVID-19-associated lung disease. If imaging is needed, CXR should be the imaging modality of choice. However, as in the case of other viral pulmonary infections, initial CXR is not indicated in immunocompetent children with mild symptoms (115), even if they require a small amount of oxygen upon admission (116). Rather, CXR should be considered in children with suspected COVID-19 presenting with moderate to severe acute respiratory symptoms (115) and in children whose clinical course doesn't follow the expected progression, or if their condition deteriorates and oxygen must be administered on day three of admission, and lastly, oxygen should be administered in children with worsening hypoxemia or who need non-invasive ventilation (116). Notably, a negative CXR neither excludes pulmonary involvement in children with laboratory confirmed COVID-19 nor indicates the absence of COVID-19 infection in cases of suspected COVID-19 infection not yet confirmed by RT-PCR (115). Thus, CXR has a poor sensitivity and specificity in the evaluation of chest involvement in children with COVID-19. Given that chest CT findings of COVID-19 are not pathognomonic in children, it is use in this population has been questioned. The American College of Radiology currently recommends against using CT as a first line screening test to diagnose COVID-19 and states that chest CT should be reserved for symptomatic hospitalized adults and children with specific clinical indications (117). The International Expert Consensus Statement on Chest Imaging in Pediatric COVID-19 Patient Management does not recommend using CT as the initial diagnostic test in children with suspected disease (115). However, CT may be considered to address a specific clinical question in the acute setting, for instance to rule out pulmonary embolism in a child with elevated D-dimer levels (115). Additionally, chest CT is extremely useful in children with a worsening clinical course and/or do not respond appropriately to supportive therapy (115). To avoid excessive radiation exposure and contamination of hospital and examination rooms and equipment, chest CT should be reserved for unstable clinically deteriorating cases, and cases in which surgery cannot be postponed. The use of LUS as a radiation-free chest investigation tool seems interesting, also given the predilection of the disease for subpleural regions (118). The procedure can be performed at bed-side with a portable device, which minimizes virus transmission, is inexpensive and can be repeated. However, the availability of experts, as well the absence of standardized interpretation criteria, limits its application (119). Table 3 summarizes abnormal chest imaging and laboratory data in children with COVID-19.

**Table 3:** Summary from current literature of abnormal chest imaging and laboratory data in children with COVID-19.

**Investigations**	**Abnormal findings**	
**Chest imaging**	**Common**	**Uncommon**
Conventional chest X-rays	Increased peri-bronchovascular markings (96) Uni/bilateral consolidations (96, 97)	Interstitial pattern, pleural effusion, pneumothorax and atelectasis (96, 97)
Chest Computed Tomography	Ground glass opacities, consolidations (with/without halo sign), tiny nodules, crazy-paving sign (102, 104)	
Lung ultrasound	B-lines with spared areas, irregular pleural lines, subpleural consolidations	Pleural effusion (109, 113, 114)
**Laboratory tests**		
Leukocytes	Normal	Increased (70, 73)
Neutrophils	Mildly decreased (70)	–
Lymphocytes	Mildly increased (70)	–
Platelets	Normal	Decreased or increased (70, 73)
Hemoglobin	Normal	Decreased (70, 94)
C-reactive protein; procalcitonin; erythrocyte sedimentation rate	Normal	Increased (70, 73, 94)
Inflammatory cytokines	Increased IL10 in severe disease	Increased IL-6 and IFN-γ in severe disease
T-lymphocyte subsets	Increased CD4^+^, CD3^+^, CD4^+^/CD8^+^ and decreased CD16^+^CD56^+^ in severe disease (70, 94)	Decreased CD8^+^ in severe disease (70, 94)
Alanine/aspartate transaminase	Normal (70, 94)	**–**
Creatinine	Normal	Decreased or increased (70, 94)
Creatine kinase	Increased (70, 94)	**–**
Lactate dehydrogenase	Normal	Increased (70, 73, 94)
D-dimer	Normal; increased only in severe forms (70, 73, 94)	**–**

## Diagnosis

The gold standard for the diagnosis of SARS-CoV-2 is RT-PCR, that entails amplification of viral RNA from a nasopharyngeal swab, tracheal aspirate or BAL specimen (120, 121). The use of bronchoscopy as a diagnostic method for COVID-19 is not recommended because it exposes both patients and healthcare staff to the risk of contamination and thus should be reserved for intubated patients when upper respiratory samples are negative (122). The specificity of the RT-PCR test seems to be about 100%, although false-positive results may be obtained due to swab contamination especially in asymptomatic patients (123). The sensitivity rate has not been clearly established, but may be ~66–80% (123). High levels of SARS-CoV-2 RNA were found in upper respiratory tract samples from an asymptomatic patient (124). It still remains to be determined how long SARS-CoV-2 RNA persists in the airways, but viral RNA has been detected weeks after exposure in patients with SARS-CoV or MERS-CoV infection (125). However, patients may not be infectious for the entire duration of virus detection because the presence of viral ribonucleic acid may not equate with a transmissible live virus. In fact, La Scola and colleagues found that the virus could not be isolated from samples collected 8 days after symptom onset (126). Unfortunately, a single negative test cannot exclude SARS-CoV-2 infection, and it may be advisable to repeat the test or to collect a deeper respiratory tract sample, such as BAL in patients known to be highly or repeatedly exposed to the virus (122).

Another test that can be used to diagnose SARS-CoV-2 is the rapid antigen test (RAT) that combines immunochromatography with an enzyme immunoassay to detect the viral nucleocapsid (N) protein (127). RAT is performed on a nasal (or nasopharyngeal) swab and the result is available in less than an hour. The RAT has a specificity of 100% and sensitivity of ~66.7%, so a positive result is usually reliable, however, given its low sensitivity, false negative results may be obtained. Although high viral loads are associated with better antigen detection rates, RAT alone is not recommended as a frontline test for COVID-19 diagnosis (127, 128).

Infections can also be diagnosed using serological methods. In this case, antibodies that are produced in response to SAR-CoV-2 and that act specifically against viral proteins are identified in blood using immunofluorescent antibody assays (129). Antibodies can also be detected with the immunochromatography method on a finger-prick blood sample. The result is obtained within a few days with the first method and within 20 min with the second. It is important to clarify that the two methods are not superimposable. Rapid COVID-19 antibody tests have a lower sensitivity than laboratory test platforms (~60%). IgG and IgM antibody test have identical sensitivity, precluding their differential use in diagnostic algorithms and levels increases in parallel during the course of infection (130). Specificity ranges from 89.2 to 100.0% (130). Importantly, the results of serological tests cannot be used to diagnose SARS-CoV-2 infection because, in acutely infected individuals, IgM might be absent up to 7–10 days after symptoms onset. Briefly, IgM antibodies appear 5–10 days after the first day of symptoms, closely followed by IgG antibodies. IgG and IgM antibodies increase during week 2 and peak in week 3, mean times for seroconversion are 9–11 days after symptom onset for total antibody, 10–12 days for IgM and 12–14 days for IgG. Levels of IgM decline from week 5 onwards and are almost non-detectable by week 7 (130, 131). However, this test provides information about previous exposure to virus as well as, if used on large scale, the disease prevalence in a population (131).

The use of saliva as easily manageable biological sample to detect SARS-CoV-2 has several advantages. In fact, it avoids the discomfort of the nasopharyngeal swab, protects health workers from exposure to the virus and overcomes the problem of the lack of nasopharyngeal swabs. In addition to rapid salivary antigen test which identifies the viral Spike protein based on the lateral flow assay (132), there is salivary RT-PCR test (133, 134). The detection of SARS-CoV-2 by RT-PCR in saliva showed a positive percent agreement of 92.5% compared to analysis in nasopharyngeal swabs, underlining that the saliva is a generally reliable specimen for the detection of SARS-CoV-2, with particular advantages for testing children (134). Salivary RT-PCR testing could be a valuable tool in mass screening strategies, especially for controlling the pandemic during the reopening period, for providing variant monitoring, and for routine testing of children as well.

## Therapeutic Strategies

No specific antiSARS-Cov-2 drugs were available at the onset of the outbreak. Several treatment strategies, mainly those used in other viral epidemics, were considered for adult patients with COVID-19. IFN and such other antivirals as lopinavir/ritonavir, anti-inflammatory drugs such as hydroxychloroquine combined or not with azithromycin, and also the immunomodulant drug tocilizumab were proposed for the treatment of COVID-19 patients. However, the clinical impact of these agents was not found to be meaningful or the risk of some of them outweighed the benefits. Indeed, these drugs are not recommended for the treatment of COVID-19. Treatment of children has been empiric-based and modeled on the experience gained in adults (135–140). A few clinical trials of COVID-19 treatment involving children are currently ongoing (141) (Table 4).

**Table 4:** Summary of anti SARS-CoV-2 therapeutic strategies in children.

**Medications results**	
***Antivirals***	
Remdesivir	Recommended/no pediatric RCTs (one open label pediatric study) (135)
Lopinavir/Ritonavir	Not recommended/no pediatric RCTs
Interferon	Not recommended/no pediatric RCTs
***Anti-inflammatory drugs***	
Hydroxychloroquine	Not recommended/no pediatric RCTs
***Immunomodulants***	
Systemic steroids	Recommended/RCTs in children (136)
Anakinra	Considered (137)/no pediatric RCTs
Tocilizumab	Considered (137)/no pediatric RCTs
Intravenous Ig	Considered (137)/no pediatric RCTs
JAKs	Non recommended/no pediatric RCTs
Convalescent plasma	Not recommended/No pediatric RCTs
***Antibiotics***	
Empiric antibiotic treatment	If evidence of bacterial co-infection (138, 139)
Azithromycin	Not recommended/no pediatric RCTs
***Anticoagulants***	
Low molecular-weight heparin	Considered (140)/no pediatric RCTs

As most children with COVID-19 are asymptomatic or have mild symptoms, supportive treatment is generally sufficient and hospitalization is not necessary. For mild and moderate cases presenting with fever, antipyretic therapy alone is indicated. Paracetamol or ibuprofen can be used according to the guidelines of fever treatment in children (142). Despite initial concerns based on theoretical postulations and some reports (143), there is no evidence that ibuprofen or non-steroidal anti-inflammatory drugs increase the risk of worse clinical outcomes in patients with COVID-19 (144), on the contrary they may be beneficial (145). Lastly, pharmacological treatment is indicated in children with severe and critical COVID-19 (146, 147) (Table 5).

**Table 5:** Summary of treatment of pediatric COVID-19, according to clinical severity (146, 147).

**Medications**	**Clinical severity of the disease**				
	**Absence of symptoms**	**Mild**	**Moderate**	**Severe**	**Critical**
**Antipyretics**					
*Paracetamol*	No	Yes, if temperature >38°C; dose: 10–15 mg/kg every 4–6 h			
*Ibuprofen*	No	Yes, if temperature >38°C; dose: 5–10 mg/kg every 6–8 h			
**Antivirals**					
*Remdesivir*	No	No	No	Yes^*^, loading dose: 5 mg/kg IV then 2.5 mg/kg/day for other 9 days.	Yes^*^, loading dose: 5 mg/kg IV then 2.5 mg/kg/day for other 9 days.
**Immunomodulants**					
Methylprednisolone^**^					
*Dexamethasone^§^*					
*Prednisone°*					
*Anakinra*^†^	No	No	No	Yes	Yes
*IV immunoglobulin^♦^*					
**Antibiotics**					
	No	Yes^Δ^, if suspected or confirmed bacterial co-infection	Yes^Δ^, if suspected or confirmed bacterial co-infection	Yes^Δ^, if suspected or confirmed bacterial co-infection	Yes^Δ^, if suspected or confirmed bacterial co-infection
**Anticoagulants**					
*Low molecular-weight heparin*	No	No	No	Yes Dose: 100–200 U/kg/day	Yes Dose: 100–200 U/kg/day

* *In hospitalized patients weighing 3.5 kg to <40 kg or aged <12 years and weighing ≥3.5 kg*.

** *Methylprednisolone 1 mg/kg IV twice a day or Methylprednisolone 30 mg/kg (max 1 g) IV pulse for 1–3 days, followed by Methylprednisolone IV/Prednisone orally, based on the severity of clinical/laboratory features*.

§ *Dexamethasone 10 mg/m^2^/dose*.

† *Anakinra 4–6 mg/kg SQ or 2 mg/kg IV (max 100 mg/dose) for 4 times a day*.

♦ *IV immunoglobulin 2 g/kg/dose (up to 70–80 g)*.

Δ *Antibiotic drug and dose according to international guidelines on pediatric infections*.

### Antiviral Drugs

Antivirals can be considered on a case-by-case basis depending on disease severity, comorbidity and side effects, and preferably if a clinical trial has confirmed the drug's efficacy and safety (146, 148). Remdesivir is the only antiviral agent currently approved for the treatment of COVID-19 patients. It is a nucleotide analog that binds to viral RNA-dependent RNA polymerase and results in premature RNA chain termination. It was well-tolerated by patients with Ebola infection (149).

The Adaptive COVID-19 Treatment Trial (ACTT-1), a randomized controlled trial of the use of remdesivir in 1,059 COVID-19 hospitalized adults, reported a faster median recovery time and decreased mortality in patients treated with remdesivir vs. patients treated with placebo (150). In April 2020, remdesivir was indicated for the treatment of severe and critical SARS-CoV-2 infection in hospitalized children in a guidance approved by the American Pediatric Infectious Diseases Society (148). In May 2020, the USA Food and Drug Administration (FDA) issued an emergency use authorization allowing the administration of remdesivir in severely compromised adults and in children hospitalized because of COVID-19, even though a warning had been issued that the co-administration of remdesivir and either chloroquine or hydroxychloroquine could reduce the antiviral activity of remdesivir (151). A phase II/III open label study involving newborns and patients up to the age of 18 years started in June 2020 to evaluate the safety, tolerability and pharmacokinetics of remdesivir (135). In November 2020, the FDA approved remdesivir for the treatment of hospitalized children with COVID-19. It can be used in children over the age of 12 years and weighing more than 40 kg. It is also available for younger children (<12 years) and for children weighing <40 kg through an FDA Emergency Use Authorization. A clinical trial evaluating the remdesivir pharmacokinetics in children is ongoing (152).

### Antibiotic Treatment

There is no evidence to support the use of antibiotics in children or adolescents with COVID-19 (138). COVID-19 patients co-infected with bacterial infection should be treated with antibiotics because secondary bacterial infections, which are mostly due to Gram-negative bacilli, are an important risk factor for mortality (138).

### Immunomodulant Treatment

Steroids, anakinra, tocilizumab, IVIG, convalescent plasma, Janus kinases, and IFNs are the agents considered in the immunomodulant treatment of pediatric COVID. Not all these agents are currently used to treat pediatric COVID-19. Steroid treatment of COVID-19 has been controversial. Initially based on the experience with SARS and MERS, systemic steroids are not recommended for the treatment of COVID-19 infection because of the risk of delayed viral clearance, worsening clinical condition and adverse events (153, 154). However, the *interim* analysis of the RECOVERY trial demonstrated that dexamethasone significantly reduced deaths in COVID-19 ventilated patients and in patients receiving only oxygen (136). At present, the IDSA Clinical Practice Guidelines recommend using dexamethasone, with or without remdesivir, to treat adult patients with critical COVID-19 (i.e., those on mechanical ventilation) and suggest administering dexamethasone, with or without remdesivir, in patients with severe COVID-19, namely those with oxygen saturation <94% and those requiring supplemental oxygen (147). Data about the safety and effectiveness of steroids for COVID-19 treatment in children are scarce. The RECOVERY trial was not sufficient to draw conclusions about steroid treatment in COVID-19 children because of the non-significant number of children enrolled and not appropriateness of the outcomes evaluated (136). Nevertheless, dexamethasone may be beneficial in children with a critical illness. Given the hyper-inflammatory state associated with COVID-19, immunomodulatory approaches, including steroids and anakinra (or tocilizumab) should be considered for children with ARDS and those with progressive deterioration of respiratory function after at least 7 days from symptom onset, as well as for children with MIS-C and/or with a markedly elevated or tendency to increasing serum levels of IL-6 and/or D-dimer and/or ferritin and/or CRP (155, 156).

Anakinra, a recombinant human IL-1 receptor antagonist that inhibits the proinflammatory cytokines IL-1α and IL-1β, and is FDA-approved for the treatment of rheumatoid arthritis and cryopyrin-associated periodic syndromes, has been used to treat MIS-C refractory to IVIG and glucocorticoids, in patients with MIS-C and macrophage activation syndrome, and in patients with contraindications to long-term glucocorticoid treatment (137). Two case series reported that anakinra reduced the need for invasive mechanical ventilation and mortality among adults with severe COVID-19 (155, 157). No clinical trials have been conducted regarding the use of anakinra in COVID-19 pediatric patients. However, two pediatric case series showed that anakinra can be safe and beneficial in children with severe disease (158, 159). However, according to National Institutes of Health (NIH) guidelines, there are insufficient data to recommend for or against the use of anakinra in adults or children with COVID-19.

Convalescent plasma consists in passive immunization using plasma collected from patients in the convalescent phase of an infection. The aim of convalescent plasma in patients with SARS-CoV-2 infection is to neutralize antibodies directed against the portions of the spike protein responsible for the binding of the virus to the ACE2 receptor (160). Several case reports and series reported that convalescent plasma stabilized or improved clinical symptoms, and laboratory and radiographic findings in COVID-19 adults affected by moderate or critical disease (161). However, in a recent RCT, convalescent plasma was not significantly better in terms of clinical status and mortality than placebo in adults affected by COVID-19 (162). Currently, little is known about the safety and efficacy of convalescent plasma therapy in children (163). Several clinical trials on the use of convalescent plasma in children are in progress (164).

Immunomodulant agents are the most widely used drugs to treat MIS-C. A review involving 662 children with MIS-C recently demonstrated that IVIG was the most frequently used medication in this population (76.4% of cases), followed by vasoactive agents (52.3%), and steroids (52.3%) (165). The IDSA Clinical Practice Guidelines suggest empiric treatment of patients with MIS-C, namely immunomodulatory agents such as high-dose IVIG, steroids, salicylates and rarely more targeted anti-inflammatory medications such as anakinra (147). Conversely, according to the USA National Institutes of Health guidelines, there is not sufficient data to recommend any of the currently available therapeutic strategies for the management of MIS-C (146). The role of antiviral medications that specifically target SARS-CoV-2 is, at present, unclear. The American College of Rheumatology Clinical Guidance for Pediatric Patients recommends a stepwise approach to immunomodulatory treatment of MIS-C, in which IVIG and/or steroids are given as first line treatment (166). Anakinra is recommended for MIS-C patients who are refractory to IVIG and/or steroids. However, immunomodulatory agents may not always be required to treat MIS-C because at least one subgroup of MIS-C patients recovered with supportive care alone (158). Considering the platelet activation, the thrombocytosis, the altered flow dynamics in abnormal coronary arteries, and the endothelial damage characteristic of Kawasaki Disease (KD), low-dose salicylate is recommended in MIS-C patients with KD features (166).

### Anti-SARS-CoV-2 Monoclonal Antibodies for the Treatment of COVID-19

Monoclonal neutralizing antibodies against SARS-CoV-2 target the SARS-CoV-2 spike protein and block virus entry into cells. The NIH Guidelines recommends using the combination of Bamlanivimab plus Etesevimab or Casirivimab plus Imdevimab to treat non-hospitalized adults and children (≥12 years and ≥40 kg) with mild to moderate COVID-19 who are at high risk of disease progression, according to High-risk Criteria of the Emergency Use Authorizations (EUA) for Anti-SARS-CoV-2 Monoclonal Antibodies (167, 168). At present, conclusive evidences about safety and efficacy of this treatment in children or adolescents are missing (169).

### Anticoagulant Treatment

COVID-19 infection has been associated with a risk of disseminated intravascular coagulation and venous thromboembolism in hospitalized children and adults. The routine use of anticoagulant treatment in children with SARS-CoV-2 infection is not recommended. In hospitalized children, pharmacologic prophylaxis and treatment with anticoagulant agents should be considered case-by-case based on laboratory findings (D-dimer >5 times upper limit of normal values) and when risk factors for thrombosis are ascertained (170). Anticoagulants are recommended in children who received anticoagulants prior to admission, or who are currently receiving extracorporeal organ support or experiencing recurrent thrombosis of venous access devices (140).

Some aspects of respiratory supportive treatment of pediatric COVID-19 warrant further consideration. Since SARS-CoV-2 infection predominantly involves the alveoli and interstitium rather than the small airways, inhaled bronchodilators would not be necessary, unlike what is recommended in viral-induced bronchiolitis. If bronchodilators or topic steroids are required, a metered dose inhaler with spacer rather than nebulization is preferred to minimize droplet spread. Patients with asthma are recommended not to discontinue inhaled or oral steroids during SARS-CoV-2 infection (171). Finally, if oxygen is prescribed for a child on the ward, low, not high, flow nasal cannula should be preferred in order to reduce the spread of droplets, unless an airborne infection isolation room is available (172).

## Comparison of SARS-CoV-2 With Other Respiratory Viruses

In the last decades a growing list of old and novel viruses have been associated to acute lower respiratory infections in children and adults. Several viruses affecting the lung have triggered epidemics, such as the HIV, the H1N1 influenza, and the coronavirus associated SARS-CoV and MERS. In the latter 2003 and 2012 coronavirus pandemics, clinical features in children were milder than in adults (173). Overall, human respiratory viruses are responsible for different clinical manifestations spanning from mild upper airway involvement to life-threatening ARDS. Of all airways infectious diseases, bronchiolitis, by far the most common in children, is caused by viruses, i.e., respiratory syncytial virus (RSV), Influenza virus, Rhinovirus, Human Metapneumovirus, Enterovirus, Coronavirus, Human Parainfluenza Virus, Adenovirus, and Bocavirus. Adenovirus infection is generally a self-limiting disease which affects 4–10% of all children, especially young children, with clinical symptoms or signs of a mild respiratory infection (174). Nevertheless, a small proportion of cases may develop bronchiolitis or pneumonia, which can be complicated by obliterans bronchiolitis and bronchiectasis, or even progress to acute respiratory failure and death. In 2001 and 2005, two novel viruses, namely Metapneumovirus and Bocavirus were detected in the airways (175). Importantly, metapneumovirus is the second leading cause of severe bronchiolitis in infancy, and high viral load and co-infection with other viral agents are risk factors for severe clinical course (176). Also bocaviruses are recognized as the etiological agents of pneumonia and bronchiolitis in children living in both cold and warm climates. Co-infection of RSV *plus* another virus, mostly rhinovirus and/or bocavirus, can be detected in children with bronchiolitis.

Recently, it was reported that also SARS-CoV-2 can cause bronchiolitis in young children (177). Therefore, even in the presence of an initially negative SARS-CoV-2 molecular testing, a high level of suspicion of COVID-19 should be maintained if other causes of bronchiolitis are unidentifiable. Symptoms and severity of COVID-19 lower airways infection in children seem no more severe than those in children with other viral infections (178). Despite this, it was recommended to check SARS-CoV-2 positive children for other viral pathogens, namely influenza A virus and metapneumovirus, as the identification of co-infections may lead to modify the diagnostic and therapeutic protocols (179). Indeed, public health measures during the COVID-19 pandemic have potential to impact transmission of other respiratory viruses, such as RSV and influenza, indicating a decline in respiratory virus detection and related hospital admissions following the implementation of COVID-19 restrictions.

## Discussion

The impressive spread of SARS-CoV-2 has led to a global emergency. Notwithstanding the large body of basic and clinical data on COVID-19 that has recently become available, various questions remain unanswered, particularly in the pediatric area. Herein, we summarize the epidemiology, pathogenesis, clinical presentation, laboratory, and chest imaging features of pediatric COVID-19 with the aim of identifying what is already known and what still needs to be explored. We are aware that a limitation of our review is the need for a quick update, given the very rapid daily growth of knowledge. Knowledge about the pathogenesis of pediatric COVID-19 is far from complete. Given the huge burden of the disease, translation of research into best practice is an urgent priority. Cross-discipline collaboration between specialists in pediatric infectious diseases and pediatric pulmonologists would probably accelerate progress in this area.

Future pediatric research should focus on:

(i) *Prevention of SARS-CoV-2 dissemination*. A growing body of evidence suggests that effective infection control interventions such as social distancing and hygiene measures are crucial to contain SARS-CoV-2 transmission and also to protect the health care system from being overloaded. Children must learn to adhere to frequent handwashing or sanitizing using alcohol-based hand-sanitizers, cough hygiene and contact avoidance behaviors even though they are less susceptible to infection than adults and have a less severe disease (27). The closure of schools as a protective measure against COVID-19 is still under discussion (180). Asymptomatic children and children with a mild disease may not pose a threat in terms of spreading the virus to other children or to adults. In fact, a recent study demonstrated that people with asymptomatic COVID-19 are infectious but might be less infectious than symptomatic cases (181). Consequently, asymptomatic children and those with mild COVID-19 are not potentially unaware vectors of infection in schools. Moreover, there is evidence of a more limited spread of SARS-CoV-2 in primary schools than in high schools, which concurs with evidence of a lower susceptibility to infection in young children than in older children and adolescents (27). Differently, SARS-CoV-2 can spread in secondary/high schools particularly when effective containment measures are not taken, for example, implementing widespread testing in schools, school closure for 24–48 h following a case in the school, quarantine of contacts of detected cases, halving the size of working groups, and mask wearing (27). In geographical areas in which these measures cannot be implemented, school closure could be considered. However, school closure and interventions such as distance learning may negatively affect children's long-term educational outcomes. Children with disabilities and those in vulnerable settings are particularly disadvantaged by school closures and distance-learning measures that do not meet their needs (180). Measures should be implemented to allow children and adolescents to attend school safely. Isolation can be unpleasant because of a reduction in social contacts however, it is extraordinarily effective in reducing the exposure of children and adolescents, whether healthy or affected by chronic lung disorders, to infections (182).

Health care institutions are major foci of disease, and the nosocomial SARS-CoV-2 transmission to health workers and hospitalized patients is a concrete possibility (183). Therefore, pediatricians must adopt rigorous measures to reduce viral transmission when assisting sick children particularly those with confirmed SARS-CoV-2 infection. Telemedicine has been installed in some health care systems to manage children and adolescents with COVID 19, and hopefully will help to reduce viral spread (184). Virus transmission during delivery is unlikely, however prevention and control measures are recommended in all neonates and particularly in pre-term newborns given their immature immune system (185).

Immunotherapy is an effective method for the prophylaxis of infectious diseases. Vaccinations have greatly decreased the burden of infectious diseases worldwide, famously leading to the eradication of small pox and to the decrease of such diseases as polio, tetanus, diphtheria, and measles. The COVID-19 vaccines currently available elicit the production of S protein neutralizing antibodies in vaccinated subjects. However, safety trials have not yet been conducted for special groups, namely children, pregnant women, and immuno-compromised patients.

(ii) *Definition of the pediatric COVID-19 phenotype*. COVID-19 has a broad spectrum of clinical presentations ranging from asymptomatic to severe deterioration of clinical conditions and even death. It is not known why the phenotypic expression of adult and pediatric COVID-19 differ. A simplistic explanation might be the lower viral load in the immune system of children vs. that of adults. The prevailing feeling is that pediatric COVID-19 might be a novel condition totally distinct from that of adults (186). There is also general concern about children with one or more underlying medical conditions (congenital heart disease, cystic fibrosis, obesity, smoke- or viral-associated airway disease, and malnutrition) who might need prolonged hospital admission and/or intensive care admission, and may have a worse outcome (186). Further studies on the pediatric COVID-19 phenotype are urgently needed to gain more insight into this aspect of the outbreak.

Once an acute COVID-19 infection has been overcome, in a subgroup of remitted adult patients there were long-term adverse effects such as persistent fatigue, diffuse myalgia, inflammation syndrome, skin rashes, depressive symptoms, and non-restorative sleep. These long-term effects of COVID-19 are commonly called long COVID. Data about long COVID in children are scarce. Ludvigsson described five children with persistent symptoms for 6–8 months after acute SARS-CoV 2 infection; predominant symptoms were fatigue, dyspnoea, joint pain, and chest pain (187). Children Long COVID can be very debilitating and lead to long school absences (187). We believe that a careful follow-up is need in children after COVID-19 to know any long-term sequelae, the mechanisms involved in the development and the proper management.

(iii) *Studies of chest imaging techniques*. Although chest imaging procedures are well-consolidated in adults in terms of radiation exposure, studies are urgently needed to identify the least invasive chest imaging techniques that have standardized interpretation criteria for children (for instance, LUS). Similarly, as residual lung fibrosis can develop after viral infection (101, 188), a longitudinal follow-up study of imaging techniques would be of remarkable value also in children with COVID-19. Chest imaging findings might also be used as a measure of efficacy of early or novel therapies in adult and pediatric COVID-19. In older children, pulmonary function testing can be considered at discharge from hospital and during follow-up (189). However, strict infection control measures must be followed during this procedure because it can be considered high-risk for viral transmission given the potential for coughing and droplet formation (190).

(iv) *Treatment*. The COVID-19 outbreak initially reduced the possibility of conducting therapeutic clinical trials with an experimental group vs. controls with no intervention or taking placebo. Moreover, children with COVID-19 often do not need treatment given their mild clinical symptoms. However, some pediatric patients progress to severe or critical acute COVID19, in which case the antiviral agent Remdesivir, and convalescent plasma and immunomodulatory agents may be beneficial. Antibiotics may be self-prescribed or unnecessarily prescribed due to theirs ease of administration, low-cost and safety. In fact, although COVID-19 and respiratory bacterial/fungal coinfection is rare, a high proportion of patients with coronavirus-associated respiratory infections have been treated with broad-spectrum empirical antibiotics (139). The potential dramatic consequence of the indiscriminate use of antibiotics in children affected by SARS-CoV-2 is increased antibiotic resistance that should not be overlooked. Fighting the threat of antibiotic resistance is a public health priority as important as the limiting of the spread of SARS-CoV-2, especially in children who have a high respiratory infection rate.

In conclusion, several aspects of pediatric COVID-19 need to be clarified. First and foremost, why are some children asymptomatic while others develop severe inflammation and even multiorgan failure? Two other aspects are equally urgent: the availability of COVID-19 vaccine programs for all children and of safe and effective treatment for the more severe forms of childhood COVID-19. Indeed, large, multicenter clinical trials are urgently needed in children and adolescents with the purpose aim of achieving encouraging results to ensure their overall well-being.

## Author Contributions

FS had full access to all of the data in the study and takes responsibility for the integrity of the data and the accuracy of the data analysis. FS, MB, AC, FF, and FC contributed substantially to the study design, data analysis and interpretation, and the writing of the manuscript. All authors contributed to the article and approved the submitted version.

## Conflict of Interest

The authors declare that the research was conducted in the absence of any commercial or financial relationships that could be construed as a potential conflict of interest.
